# Surrogate Endpoints for Late Kidney Transplantation Failure

**DOI:** 10.3389/ti.2022.10136

**Published:** 2022-05-20

**Authors:** Maarten Naesens, Klemens Budde, Luuk Hilbrands, Rainer Oberbauer, Maria Irene Bellini, Denis Glotz, Josep Grinyó, Uwe Heemann, Ina Jochmans, Liset Pengel, Marlies Reinders, Stefan Schneeberger, Alexandre Loupy

**Affiliations:** ^1^ Department of Microbiology, Immunology and Transplantation, KU Leuven, Leuven, Belgium; ^2^ Department of Nephrology and Medical Intensive Care, Charité Universitätsmedizin Berlin, Berlin, Germany; ^3^ Department of Nephrology, Radboud University Medical Center, Nijmegen, Netherlands; ^4^ Department of Nephrology and Dialysis, Medical University of Vienna, Vienna, Austria; ^5^ Department of Surgical Sciences, Sapienza University of Rome, Rome, Italy; ^6^ Paris Translational Research Center for Organ Transplantation, Hôpital Saint Louis, Paris, France; ^7^ University of Barcelona, Barcelona, Spain; ^8^ Department of Nephrology, Technical University of Munich, Munich, Germany; ^9^ Centre for Evidence in Transplantation, Nuffield Department of Surgical Sciences, University of Oxford, Oxford, United Kingdom; ^10^ Department of Internal Medicine, Erasmus MC Transplant Institute, University Medical Center Rotterdam, Rotterdam, Netherlands; ^11^ Department of General, Transplant and Thoracic Surgery, Medical University of Innsbruck, Innsbruck, Austria; ^12^ Paris Translational Research Center for Organ Transplantation, Hôpital Necker, Paris, France

**Keywords:** rejection, outcome, graft function, conditional marketing authorization, iBox

## Abstract

In kidney transplant recipients, late graft failure is often multifactorial. In addition, primary endpoints in kidney transplantation studies seek to demonstrate the short-term efficacy and safety of clinical interventions. Although such endpoints might demonstrate short-term improvement in specific aspects of graft function or incidence of rejection, such findings do not automatically translate into meaningful long-term graft survival benefits. Combining many factors into a well-validated model is therefore more likely to predict long-term outcome and better reflect the complexity of late graft failure than using single endpoints. If conditional marketing authorization could be considered for therapies that aim to improve long-term outcomes following kidney transplantation, then the surrogate endpoint for graft failure in clinical trial settings needs clearer definition. This Consensus Report considers the potential benefits and drawbacks of several candidate surrogate endpoints (including estimated glomerular filtration rate, proteinuria, histological lesions, and donor-specific anti-human leukocyte antigen antibodies) and composite scoring systems. The content was created from information prepared by a working group within the European Society for Organ Transplantation (ESOT). The group submitted a Broad Scientific Advice request to the European Medicines Agency (EMA), June 2020: the request focused on clinical trial design and endpoints in kidney transplantation. Following discussion and refinement, the EMA made final recommendations to ESOT in December 2020 regarding the potential to use surrogate endpoints in clinical studies that aim to improving late graft failure.

## Introduction

Key primary endpoints in kidney transplantation are recipient death, graft failure, biopsy-confirmed acute rejection, and graft (dys)function. These endpoints have clear roles in research that aims to improve short-term clinical outcomes after transplantation, and they are also the efficacy endpoints used most often in clinical trials (1). However, as improvement in short-term graft survival (by inhibition of early rejection) does not translate into an equally long-term improvement in graft survival, and as graft failure is rare in the early years following transplantation, better predictors of long-term graft outcome are needed for use in randomized controlled trials (RCT).

If conditional marketing authorization could be considered for therapies that aim to improve long-term outcomes [see Naesens et al., this issue (2)], then the surrogate endpoint for graft failure (i.e., loss of graft function; excluding death with a functioning graft) for use in RCT needs clearer definition. A good surrogate endpoint should fulfill four criteria: 1) The disease process is sufficiently understood; 2) The surrogate endpoint has biologic plausibility; 3) The strength of the consistency supports the relationship between the surrogate marker and outcome; 4) Treatment effects on the surrogate endpoint predict treatment effects on the clinical outcome of interest (Table 1). In addition, the acceptability of a surrogate endpoint for conditional marketing authorization of new therapies also depends on a benefit–risk evaluation and/or public health aspects, such as a serious life-threatening disease with no other therapeutic option, difficulties with studying the (rare or delayed) clinical endpoint, and the availability of a large safety database (2).

**TABLE 1:** Criteria for a valid surrogate endpoint, applied to potential surrogate endpoints in kidney transplantation.

Criterion	Proteinuria	DSA	eGFR + proteinuria combined	Chronic graft histology	iBox score
Disease process (graft failure) sufficiently understood	Confirmed	Confirmed	Confirmed	Confirmed	Confirmed
Biologic plausibility	Confirmed	Confirmed	Confirmed	Confirmed	Confirmed
Strength of consistency supporting relationship between surrogate marker and outcome	Confirmed	Confirmed	Not confirmed	Not confirmed	Confirmed
Treatment effects on surrogate endpoint predict treatment effects on clinical outcome of interest	Not confirmed	Not confirmed	Not confirmed	Not confirmed	Not confirmed

DSA, donor-specific antibody; eGFR, estimated glomerular filtration rate.

## Definition and Causes of Graft Failure

Graft failure/loss of graft function is defined as return to dialysis or pre-emptive re-transplantation. Death of the recipient with a functioning graft is typically a primary safety endpoint, but we do not recommend including this in a surrogate endpoint for kidney transplantation outcome because of the wide variety of underlying causes of death observed (e.g., malignancy, infection, cardiovascular disease), lack of relation to graft functional status, and very different risk factors compared with those for graft failure (3, 4). These causes of death are often influenced by immunosuppression (5).

Furthermore, death with a functioning graft is a competing risk to loss of graft function, as is also the case in chronic kidney disease (CKD). In CKD, censoring for death increasingly overestimated the risk of kidney failure over time from 7% at 5 years to 19% at 10 years, especially in people at heightened risk of death (6). Although it could be anticipated that this is also relevant in kidney transplantation, the impact of this competing risk on the accuracy of death-censored graft failure risk is poorly established.

Definitions of all-cause and overall graft failure are discussed elsewhere in this Special Issue (2); of note, in this document, “graft failure” denotes loss of graft function, not overall graft failure (which includes patient death as a reason for graft failure). Given that late graft failure (excluding death with a functioning graft) is often multifactorial (4), it is difficult to predict such failure accurately with a single marker; a composite marker may more fully reflect the heterogeneity. The most important causes of graft failure are acute or chronic T-cell mediated rejection (TCMR), antibody-mediated rejection (AMR), nonspecific chronic injury due to nephron loss (drug toxicity, metabolic and urological factors), calcineurin inhibitor toxicity, infection, and other medical events (cardiorenal problems, vascular disease, malignancy, postrenal causes) (7), as well as occurrence or recurrence of original kidney disease. Consequently, the following markers are associated with heightened risk of late graft functional decline and failure: measured glomerular filtration rate (GFR); estimated (e)GFR, slope of eGFR trajectory, and eGFR change; CKD stage; proteinuria; *de novo* (*dn*) donor-specific antibodies (DSA); AMR histology; interstitial fibrosis and tubular atrophy (IFTA); and transplant glomerulopathy (TG) (8, 9).

## Single Markers as Surrogate Endpoint

Single surrogate markers of graft function may not fully reflect the complexity of graft failure and death in kidney transplantation because some background (donor or recipient) risk factors—such as age and pre-existing immunological risk, including pre-transplant DSA—also affect outcome and graft-function markers. Late graft failure is more complex than renal failure resultant from native kidney disease because of competing risks involved at different time points. For example, the ZEUS trial (phase III randomized trial of cyclosporine continuation vs. switch to everolimus at 4.5 months post-transplant) showed a slightly better GFR (the primary endpoint), but higher rates of DSA and AMR (with absence of effect on graft failure and increased risk of graft failure) in patients who developed *dn*DSA (7, 10). Furthermore, creating too stringent a definition of factors such as change in eGFR would require studies with long duration and large patient populations, which are difficult to achieve (11).

Conversely, considering only minor changes in a surrogate endpoint, such as eGFR or transplant glomerulopathy, increases the risk of error. For example, in histological terms, new or worsening transplant glomerulopathy could be considered as a surrogate endpoint in clinical trials, but the intrinsic heterogeneity of this pathology and varied data on its association with death-censored graft survival (12) make it difficult to translate findings into predictions for late events. In addition, this parameter has neither been used, nor accepted, by health authorities.

Combining multiple factors into a well-validated model is therefore more likely to predict long-term outcome (and better reflect the complexity of late graft failure) than using single endpoints or combining few factors. Relatively short-term improvements in such a complex score ideally would translate into long-term improvements in graft survival. It is also important that a valid surrogate marker for a well-understood disease process should have biological plausibility and a consistent relationship with outcome. Finally, treatment effects that change the surrogate marker should also have impact on clinical outcome.

Here we review the putative surrogate endpoints, including composite endpoints for predicting long-term graft outcome (excluding death with a functioning graft), focusing on eGFR, proteinuria, histological lesions, DSA, and complex scoring systems (Table 1).

### GFR and eGFR

For in-depth discussion on the association between kidney function and graft failure, methodology for measuring kidney function and its validity as a primary endpoint for clinical trials, see Hilbrands et al. (13).

Because graft failure is intrinsically defined by functional parameters such as dialysis reinitiation or repeat transplantation, graft functional assessment is directly related to the true endpoint, graft failure. Any intervention that stabilizes long-term graft function will inherently decrease the incidence of graft failure, therefore graft function is a direct measure of graft failure.

Predicted graft survival based on 12-months eGFR correlates with observed graft survival (14); consequently, eGFR alone is potentially interesting as a surrogate marker for long-term graft failure. This parameter was applied in the only relatively recent organ transplantation study to show improved long-term outcome using a new treatment (belatacept) (15, 16). However, declining eGFR is a late and insensitive marker for late graft failure in heterogeneous populations (17). The initial injury processes contributing to late graft failure are subclinical, and not reflected by early decline in renal function. Consequently, the long-term predictive value of measures of early post-transplantation renal function is limited (17); such measures (including serum creatinine values and use of eGFR) are discussed elsewhere in this supplement (13).

Additional graft injuries may develop slowly over time: declining renal function is the ultimate consequence of nephron loss but does not capture causes of nephron injury. Also, compensatory hyperfiltration may obscure initial damage. Moreover, the static absolute level of eGFR is also related to donor (e.g., age, brain death, hypotension) and transplant (e.g., ischemia/reperfusion) factors that might reduce the number of functioning nephrons at transplantation; using a single eGFR measurement as a surrogate endpoint would not take these into account.

Clearly, GFR has limitations as a surrogate for late graft failure, since in the first year after transplantation it fails to capture ongoing disease processes that lead to late graft failure. Sensitive tools that better reflect the heterogeneity in causes of late graft failure are required.

### Proteinuria

In CKD research there is increasing interest in using degree of proteinuria as a surrogate endpoint: the proteinuria level directly relates to the underlying glomerular disease process, and strongly correlates with progression to end-stage renal disease (18, 19). Proteinuria is routinely measured after kidney transplantation (20, 21); severe proteinuria in the nephrotic range often reflects structural damage to the nephron and is therefore associated with graft outcome (8, 22, 23). Histological signs of structural abnormality are TG, microcirculatory inflammation, and *dn* or recurrent glomerular disease (8), all of which are important causes of late graft failure.

Post-transplantation proteinuria thus tends to indicate poor prognosis, independent of graft function as assessed by eGFR (8, 24, 25), but may also reflect disease processes beyond renal function. Similar to general-population studies, an analysis that prospectively adjudicated cardiovascular events showed that albuminuria was strongly associated not only with graft failure, but also with cardiovascular events and mortality (25). Proteinuria alone has not widely been included as a surrogate endpoint in interventional studies of kidney transplantation and correcting post-transplantation proteinuria has not been proven to reduce the rates of long-term graft failure in studies of antihypertensive medication use in transplant populations (26–30). Conversely, studies with mTOR inhibitors revealed increases in proteinuria that did not translate into increased rates of long-term graft failure (7, 31).

### Donor-Specific HLA Antibodies

Since the early days of clinical kidney transplantation, it has been recognized that antibodies directed against non-self human leukocyte antigen (HLA) could be extremely relevant for graft outcomes. A seminal study described the key features and potential impact of alloantibodies in transplantation, demonstrating that immediate catastrophic graft failure is more likely to happen in multiparous female patients or in people receiving second transplants, and is seen in up to 80% of cases where there was a “positive crossmatch” (i.e., reactivity of recipient serum against donor cells) (32). The researchers advocated that demonstration of preformed cytotoxic antibodies against the graft (“sensitization”) contraindicates allocation of a proposed graft to the transplant candidate. This recommendation was rapidly adopted and, aside from patients successfully desensitized by empirical approaches, remains rigidly enforced, using complement-dependent cytotoxicity crossmatching (CDC-XM) (32, 33).

With time, however, it became clear that CDC-XM lacks sensitivity for detecting circulating DSA: not all clinically significant pre-transplant DSA are identified. This led to the development of sensitive solid-phase tests, such as Luminex^®^ single-antigen bead (SAB) assays (R&D Systems Inc., Minneapolis, MN, United States), which detect low-level DSA when the CDC test is negative. The definition of HLA antibody specificity by SAB assays added complexity to transplant risk stratification, by revealing extensive heterogeneity in the pathogenic potential of HLA-DSA. It is now well established that patients with pretransplant DSA detected by SAB, even with a negative CDC crossmatch, are at substantial risk of AMR and graft failure (34–37). Flow cytometry cross-matching adds additional insight into the actual immunologic risk for such patients (38).

The role of circulating anti–HLA-DSA is increasingly recognized as a major contributing factor to AMR and long-term graft failure (39–41). However, the occurrence of newly formed *dn*DSA after transplantation further increases the risk of graft failure (42–47), and complement-fixing DSA are particularly associated with graft rejection and failure (48). Some immunosuppressants (e.g., belatacept) appear to inhibit the development of *dn*HLA-DSA (16), while others (e.g., mTOR inhibitors) can be associated with a higher frequency of *dn*HLA-DSA (49). Importantly, under-immunosuppression and patient nonadherence are important risk factors for *dn*HLA-DSA development (50).

The STAR working group, a collaboration between the American Society for Histocompatibility and Immunogenetics and the American Society of Transplantation (51), made recommendations on the definitions and utilization of HLA diagnostic testing. In Europe, the European Federation for Immunogenetics publishes standards for histocompatibility and immunogenetics testing (52). Limitations of Luminex SAB assays that have been described include their semiquantitative nature, the prozone effect, test variability, and the need for arbitrary cut-off values to determine positivity. There are also technical challenges; for example, thresholds for DSA positivity are poorly defined and inconsistent, with European immunogenetics groups proposing mean fluorescence intensity (MFI) cut-off values of >3,000 or >5,000 MFI (53) and US groups proposing 1,400 MFI, which requires validation (51). A consistent definition of such a cut-off value, to indicate presence or absence of HLA antibodies, is crucial if DSA is to be considered as a single endpoint in RCTs. In addition, SAB MFI should not be used as a quantitative assay since it has a relatively high coefficient of variation (51). Thus, current technology cannot determine antibody titers or the clinical and biological relevance of positive test results (51, 54). In addition, although pretransplant DSA and *dn*HLA antibody development are strongly associated with AMR and graft failure (43, 55–60), no studies show that interventions affecting DSA levels or specificities after transplantation predict long-term improvement in graft survival rates (Table 2) (54, 61–63).

**TABLE 2:** Association between changes in DSA and graft outcome in kidney transplantation RCTs. No studies show that interventions that affect DSA predict long-term graft outcomes (55, 61–63).

Study	Setting and intervention	Effect on DSA	Effect on graft outcome
Bray et al., 2018 (55)	Belatacept vs. cyclosporine in the BENEFIT and BENEFIT-EXT studies	Significantly lower risk of *dn*DSA development and lower MFI of these DSA	Significantly better overall graft failure but equal death-censored graft failure and AMR risk
Moreso et al., 2018 (61)	IVIG + rituximab for chronic AMR	No change in immunodominant DSA-MFI between baseline and 1 year	No change in renal function assessed by eGFR (underpowered study)
Eskandary et al., 2018 (62)	Bortezomib vs. placebo for treatment of late AMR	No change in DSA-MFI	No change in renal function assessed by eGFR or graft failure
Sautenet et al., 2016 (63)	Rituximab vs. placebo for AMR	Significantly decreased DSA-MFI	No effect of the intervention on graft function or graft survival (underpowered study)

AMR, antibody-mediated rejection; dn, *de novo*; DSA, donor-specific antibodies; eGFR, estimated glomerular filtration rate; IVIG, intravenous immunoglobulin; MFI, mean fluorescence intensity; RCT, randomized controlled trial.

*Post hoc* analyses of the BENEFIT and BENEFIT-EXT studies (phase III randomized trials of belatacept vs. cyclosporine) showed significant reductions in the risk of *dn*DSA occurrence (55) and best overall graft survival rates. However, numbers were too small to demonstrate that these effects were mediated through improved death-censored graft survival or decreased risk of AMR. In contrast, data from mTOR inhibitor conversion studies showed higher rates of DSA and AMR in groups treated with mTOR inhibitors, but during the observation period no overall effect on graft survival was noted (64, 65), although follow-up was short, and DSA status was often missing (65). Finally, although the RITUX ERAH RCT (randomized trial of rituximab vs. placebo in addition to plasma exchange, intravenous immunoglobulin and corticosteroids for the treatment of AMR) showed an effect of rituximab on DSA-MFI that did not translate into improved graft function or survival rate, this study was underpowered, so firm conclusions could not be made (66).

As identified in a systematic review (67), therapeutic strategies eliminating *dn*DSA, tested in RCTs that are sufficiently powered to assess long-term graft outcomes, are needed. Case series suggest that “impossible” transplants become possible with pre-transplant desensitization of HLA antibodies (67), but this does not validate HLA-DSA levels or specificities as surrogates for long-term outcome.

In summary, only the development of *dn*HLA-DSA with a clear MFI signal could be a meaningful surrogate endpoint that is strongly associated with adverse outcomes such as AMR and graft failure. While *dn*DSA development is clearly associated with immunosuppression, patient nonadherence (especially under-immunosuppression) may also play a role. The development of *dn*HLA-DSA has not been formally tested or validated as a surrogate endpoint for studies that aim to reduce graft failure because of AMR. In addition, as graft failure is heterogeneous and often no HLA-DSA are involved, *dn*DSA occurrence is insufficient as a surrogate for late graft failure by causes other than AMR.

## Combined Functional Markers

The risk of adverse outcomes at a given eGFR certainly increases with higher levels of albuminuria. In addition, integrating proteinuria and eGFR assessment is a good predictor of graft outcome (24, 25); studies also demonstrate an independent association between graft outcome and eGFR or proteinuria (8, 68).

Although potentially interesting as surrogate marker, the performance of a model that integrates proteinuria and eGFR has not been further validated in transplantation (25). However, whether the combination of eGFR and proteinuria could be considered as a primary (rather than surrogate) endpoint in kidney transplantation, as it is in CKD, warrants further discussion. Indeed, in CKD, the KDIGO guideline on prognostication based on integration of eGFR and albuminuria is an accepted surrogate for outcome in clinical trials, but the European Medicines Agency (EMA)’s CHMP guideline for primary prevention (69) proposed two primary efficacy endpoints: prevention or slowing of decline in the level of renal function (defined as either time to occurrence of CKD 3 or incidence rate of CKD ≥3); and clinically meaningful and stable difference in GFR failure rate with or without prevention of proteinuria/albuminuria. A similar primary endpoint could be considered in kidney transplantation, and the US Food and Drug Administration already follows this approach (70). However, no RCT has been undertaken to demonstrate that changes in such a composite functional endpoint predict changes in long-term graft survival rates.

## Composite Scores

Late graft failure (excluding death with a functioning graft) is a highly multifactorial state (4) that relates not only to early graft function, but also to subclinical injury processes including progressive IFTA or TG, drug toxicity, infections, medical events, recurrent disease, microvascular injury, and circulating DSA. Graft function is also highly dependent on pre-transplant donor/recipient risk factors (e.g., age, sex, delayed graft function), which further complicate the value of interpreting a single measurement of function as a surrogate for long-term outcome: studies show independent associations between these factors and graft failure in multivariate models (Table 3) (8, 9).

**TABLE 3:** HR (multivariate models) for graft failure according to graft histology, renal function, and proteinuria at time of biopsy, adjusted for donor age and time after transplantation (8,9).

Parameter		Adjusted HR (95% CI)	*p* value
Naesens et al., 2016 (N = 1,335 indication biopsies) (8)			
Proteinuria at time of biopsy	0.3–1.0 vs. <0.3 g/24 h	1.14 (0.81–1.60)	0.50
	1.0–3.0 vs. <0.3 g/24 h	2.17 (1.49–3.18)	<0.001
	>3.0 vs. <0.3 g/24 h	3.01 (1.75–5.18)	<0.001
eGFR at time of biopsy	30–45 vs. >45 ml/min/1.73 m^2^	1.76 (0.59–5.30)	0.31
	15–30 vs. >45 ml/min/1.73 m^2^	5.53 (1.99–15.4)	0.001
	<15 vs. >45 ml/min/1.73 m^2^	11.7 (4.17–33.0)	<0.001
Microcirculation inflammation	g + ptc ≥2 vs. <2	1.36 (0.97–1.91)	0.07
IFTA grade	Banff grade 1 vs. 0	1.82 (1.25–2.64)	0.002
	Banff grade 2–3 vs. 0	3.45 (2.34–5.07)	<0.001
Transplant glomerulopathy	Banff grade 1 vs. 0	1.00 (0.55–1.82)	0.99
	Banff grade 2–3 vs. 0	1.83 (1.11–3.04)	0.02
*De novo*/recurrent glomerular disease	Present vs. absent	1.35 (0.84–2.19)	0.22
Polyomavirus-associated nephropathy	Present vs. absent	5.51 (3.06–9.92)	<0.001
Loupy et al., 2019 (N = 3,941 patients) (9)			
Time from transplant to evaluation (years)		1.08 (1.02–1.14)	0.0051
eGFR (mL/min/1.73 m^2^)		0.96 (0.95–0.96)	<0.0001
Proteinuria (log)		1.51 (1.40–1.63)	<0.0001
IFTA	0/1	—	
	2	1.14 (0.918–1.424)	
	3	1.39 (1.083–1.773)	0.0311
Microcirculation inflammation (g + ptc)	0–2	—	
	3–4	1.45 (1.121–1.876)	
	5–6	1.83 (1.240–2.706)	0.0010
Interstitial inflammation and tubulitis (i + t)	0–2	—	
	≥3	1.34 (1.061–1.684)	0.0136
Transplant glomerulopathy (cg)	0		
	≥1	1.47 (1.133–1.895)	0.0036
Anti–HLA-DSA MFI	<500	—	
	≥500 to 3,000	1.25 (0.965–1.606)	
	≥3,000 to 6,000	1.72 (1.115–2.659)	
	≥6,000	2.05 (1.472–2.860)	0.0001

cg, transplant glomerulopathy; CI, confidence interval; DSA, donor-specific antibodies; eGFR, estimated glomerular filtration rate; g, glomerulitis score; HLA, human leukocyte antigen; HR, hazard ratio; i, interstitial; IFTA, interstitial fibrosis and tubular atrophy; MFI, mean fluorescence intensity; ptc, peritubular capillaritis score; t, tubulitis score.

A systematic review evaluated models developed to predict graft failure in kidney transplantation recipients (71). Fourteen studies used predictors that were measured after transplantation; few studies integrated graft functional data such as proteinuria (n = 5) or serum creatinine/eGFR (n = 12), and none evaluated histology as part of the composite prediction model. Nineteen studies reported on the validity of the model in external datasets, several of which warrant in-depth assessment of their potential usefulness as surrogate endpoints for long-term graft failure excluding death with a functioning graft (14, 72–77); key features of these publications are listed in Table 4. Another study suggested a composite method for predicting graft failure; but because it included recipient death, it is less appropriate than other approaches as a potential surrogate endpoint for death-censored graft failure (78, 79).

**TABLE 4:** Value of composite scores as surrogacy for long-term graft survival (9, 14, 72–77).

Study	Kasiske et al., 2010 (72)	Foucher et al., 2010 (73)	Moore et al., 2011 (74)	Schnitzler et al., 2012 (14)	Shabir et al., 2014 (75); Gonzales et al., 2016 (76)	Gonzales et al., 2016 (76)	Prémaud et al., 2017 (77)	Loupy et al., 2019 (9)
Parameter	USRDS Risk Prediction Tool	KTFS	LOTESS Composite Risk Score	USRDS Predictive Model	Birmingham Risk Score	Birmingham-Mayo Histology-Based Model	AdGFS	iBox Risk Prediction Score
Development set	USRDS registry data (N = 59,091)	Multicentre French registry (N = 2,169)	Multicentre national cohort study (N = 2,763)	USRDS registry data (N = 87,575)	Single-center UK data (N = 651)	Single-center US data (N = 1,465)	Single-center French data (N = 664)	French multicentre cohort (N = 4,000)
External validation	No	Yes (N = 317)	Yes (single UK center; N = 731)	No	Yes (2 European centers (N = 736, N = 787) and 1 Canadian center (N = 475); 1 US center N = 1,465)	No	Yes (2 other French centers; N = 896)	Yes; N = 3,557 (2,129 patients in 3 European centers; 1,428 in 3 North American centers)
Prediction time point	12 months post-transplant	12 months post-transplant	Variable time after 12 months post-transplant	12 months post-transplant	12 months post-transplant	12 months post-transplant	Time adjusted (only for ‘rejection’)	Time adjusted
Outcome parameter	Overall graft failure at 5 years after transplantation	Death-censored graft failure at 8 years	Overall graft failure and death-censored graft failure over time; follow-up time not specified	Overall graft failure beyond 1 year post-transplant, up to 9 years	Overall graft failure and death-censored graft failure at 5 years post-transplant	Overall graft failure and death-censored graft failure at 5 years post-transplant	Death-censored graft failure beyond 2 years post-transplant, up to 10 years	Death-censored graft failure over time post-transplant, up to 7 years
Pre-transplant factors included in the model	Recipient age	Recipient sex	Recipient age	A large array of donor and recipient demographic factors (N = 20)	Recipient age	Recipient age	Donor age Pre-transplant non-DSA HLA antibodies	Yes, adjusted for all relevant factors
	Recipient race	Recipient age	Recipient sex		Recipient sex	Recipient sex		
	Insurance	# Previous transplantations	Recipient race		Recipient race	Recipient race		
	Cause of ESRD	Donor creatinine						
Post-transplant factors included in the model	eGFR at 12 months Hospitalization	Serum creatinine Acute rejection Creatinine at 3 months 24-h proteinuria	eGFR at 12 months eGFR evolution Acute rejection Serum urea at 12 months Serum albumin	eGFR at 12 months Acute rejection within first year	Acute rejection eGFR Serum albumin UACR	Acute rejection eGFR UACR Black ethnicity Glomerulitis score Tubular atrophy score	Serum creatinine Proteinuria *dn*DSA Serum creatinine trajectory Acute rejection	Time post-transplant eGFR Proteinuria Histology (IFTA, microcirculation inflammation, TG) DSA-MFI
Prognostic accuracy	C-statistic 0.65–0.78	ROC AUC 0.78 (0.73–0.80)	C-statistic 0.83 for death-censored graft failure; 0.70 for overall graft failure	Not reported	C-statistic 0.78–0.90 for death-censored failure; 0.75–0.81 for overall graft failure	C-statistic 0.90 for death-censored failure; 0.81 for overall graft failure	C-statistic at 10 years post-transplant 0.83 (0.76–0.89)	C-statistic 0.81 in development cohort, 0.81 in European validation cohort, 0.80 in US validation cohort
Calibration	Good	Not assessed	Good	Good	Good	Good	Good	Good
Limitations	No external validation set No data on DSA No data on proteinuria Prognostic accuracy moderate	Small validation set Validity not tested in other countries No data on DSA No data on rejection phenotype Limited prognostic accuracy	Small validation set Validity not tested in other countries No data on DSA No data on rejection phenotype Prediction time point variable	No external validation set No data on DSA No data on proteinuria No data on rejection phenotype	No data on rejection phenotype No data on DSA	No external validation set Data on DSA did not improve the model	Small validation sets and validity in other countries not tested Not tested in living donors or patients with pre-transplant DSA	Not yet prospectively implemented in an RCT
Tested in randomized trial data?	No	No	No	Yes, but calibration and validity as surrogacy for improved outcome by the intervention was not tested	No	No	No	Yes; validation in 3 RCTs; association with improved outcome not confirmed given lack of efficacy of the intervention

AdGFS, adjustable score for prediction of graft failure; dn, *de novo*; DSA, donor-specific antibodies; eGFR, estimated glomerular filtration rate; ESRD, end-stage renal disease; IFTA, interstitial fibrosis and tubular atrophy; KTFS, kidney transplantation failure score; LOTESS, long-term efficacy and safety surveillance; MFI, mean fluorescence intensity; RCT, randomized controlled trial; TG, transplant glomerulopathy; UACR, urine albumin to creatinine ratio; USRDS, United States Renal Data System.

In the study by Kasiske et al. (72), eGFR at 1 year was the only functional value included in the final model for prediction of 5-years graft failure, along with baseline recipient criteria and hospitalization within the first year following transplantation. However, this analysis was performed on a large registry (USRDS) that lacked crucial information on several clinical parameters. Furthermore, although the model showed good calibration, no independent validation was performed, and the impact of therapeutic interventions that aimed to reduce long-term graft failure was not tested. Moore et al. (74) restricted post-transplant factors in the model to eGFR and eGFR evolution, but nevertheless reached adequate discrimination and calibration for death-censored graft failure. External validation was restricted to a single center, and again the impact of therapeutic interventions was not evaluated. Importantly, the risk scores derived and tested in this study offered no prognostic superiority over basic metrics, such as eGFR or recipient age in isolation (74).

Foucher et al. proposed a clinical scoring system, built on the French DIVAT registry (3). The score was constructed at 1 year post transplantation, for prediction of graft failure at 8 years, and reached a C-statistic of 0.78. External validation was performed, but in a small dataset (n = 317). Other limitations included limited exportability, restriction to French transplant centers, and no inclusion of data on DSA and rejection subtypes or histological lesions. In addition, this score was built on observations at only one time point. The potential of this prognostic score to be used as surrogacy for long-term graft failure was not tested in any RCT aiming to improve long-term outcome.

The first study to implement a previously developed risk score, in the context of a RCT aiming to improve long-term graft outcome, analyzed data from the USRDS registry (1995–2004) (14). Prediction models for all-cause graft survival were applied to participants in the BENEFIT and BENEFIT-EXT studies (phase III randomized trials of belatacept vs. cyclosporine), to determine whether the model could be used as a surrogate endpoint for late graft failure. Predicted and observed all-cause graft failures were well calibrated in standard- and expanded-criteria donor kidneys, as evaluated in the development cohort. Although data on model accuracy were lacking, aspects including eGFR and donor/recipient characteristics revealed a striking concordance between predicted and observed graft survival rates, when evaluated for 1-year outcome (14). However, when predicted survival estimates for 7 years post transplantation were compared with actual outcomes (16, 80), the predicted versus observed overall graft survival for the less-intensive group was 73.9 vs. 87.2%, and for the cyclosporine group was 69.0 vs. 78.3%. This illustrates that the calibration of the model for predicting longer-term survival was perhaps less than anticipated, which might be explained by the model being built on data obtained in an older era. As the surrogacy of the model established at 1 year for long-term graft failure was not directly confirmed, it is questionable whether it provides sufficient accuracy and calibration for use as a complex surrogate endpoint in future RCTs (14).

Shabir et al. developed a prediction model for 5-years graft failure using data from a single UK center, at 12 months post transplantation (75). The resultant risk scores were evaluated for prognostic utility (discrimination, calibration, and risk reclassification) in three independent cohorts in Europe and Canada. Recipient age, sex, and race; acute rejection rate; eGFR; serum albumin level; and urine albumin/creatinine ratio were included in scores for death-censored and overall graft failure. The rejection subtype was not further specified. In the validation cohorts, these scores showed good-to-excellent discrimination for death-censored transplant failure and moderate-to-good discrimination for overall transplant failure. Both scores demonstrated good calibration. Compared with eGFR in isolation, application of the scores resulted in statistically significant and clinically relevant risk reclassification for death-censored transplant failure [net reclassification improvement (NRI) 36.1–83.0%; all *p* < 0.001] and overall transplant failure (NRI 38.7–53.5%; all *p* < 0.001). Compared with the USRDS-based calculator, significant and relevant risk reclassification for overall transplant failure was seen (NRI 30.0%; *p* < 0.001) (75).

These scores have been externally validated (76): the risk model integrated 1-year histological and antibody data for prediction of graft failure at 5 years post transplantation in a single-center study (n = 1,465). The Birmingham Risk Score performed well, with good discrimination for recipients with or without graft failure 5 years after transplantation for both overall and death-censored graft failure (C-statistic 0.78 and 0.84, respectively), although this score has not been evaluated in an RCT designed to assess improvement of long-term graft outcome. Adding glomerulitis and interstitial fibrosis data to the Birmingham Risk Score improved the C-statistic for death-censored graft failure from 0.84 to 0.90, with further improved calibration and significant reclassification.

Decision-curve analyses aimed to determine how risk prediction could be improved when histological data were added to the clinical risk model proposed by Shabir et al. (75). However, this expanded model has not been independently validated and the impact of therapeutic interventions has not been evaluated. Prémaud et al. proposed a composite adjustable score for prediction of graft failure (AdGFS) using a conditional survival-tree analysis, undertaken using variables from patients transplanted between 1984 and 2011 in a French center (77). The analysis was based on serum creatinine and proteinuria at 12 months, *dn*DSA, serum creatinine cluster (creatinine value trajectories within the first year), acute rejection, donor age, and pre-transplant non-donor-specific HLA antibodies. Predictive performance of the AdGFS was good and the accuracy of the score at predicting graft failure remained high in the validation dataset, and in the external dataset (consisting of 896 patients from two other French centers, transplanted between 2002 and 2010). However, the study had limitations: the cohort did not represent current practice, there was no evaluation of the AdGFS response to therapies that aim to improve long-term graft outcome, validity in living donor kidney transplants and in recipients with pretransplant DSA was not tested, and data on DSA were lacking. In addition, international validation has not been performed.

### iBox

Loupy et al. developed the largest and only specifically designed multivariate model that predicts long-term death-censored graft failure: the iBox model was created after a study was undertaken in which parameters were collected from day of transplantation, to provide a holistic appraisal of potential risk factors (9). Their data showed that, among 7,557 kidney transplant recipients, 1,067 grafts failed (14.12%) in a median post-transplant follow-up of 7.12 years [interquartile range (IQR) 3.51–8.77] (9). In the derivation cohort, eight functional, histological, and immunological prognostic factors were found to be independently associated with death-censored graft failure. These were then combined into a risk prediction score that included the following parameters, in order of importance: eGFR; proteinuria:creatinine ratio; structural markers [Banff IFTA grade, microcirculation inflammation (Banff g + ptc), TG (Banff cg score), interstitial inflammation, and tubulitis (Banff i + t)]; MFI of the immunodominant HLA-DSA, and time from transplant to risk evaluation. The risk prediction score exhibited accurate calibration and discrimination (0.81 derivation and 0.80–0.81 in validation cohorts) (9). The performance of this multivariate model was validated in cohorts from three European and three North American centers (9). Importantly, testing the iBox model involved unselected patient cohorts, covering all potential clinical scenarios.

The iBox model was accurate when assessed independently of time since transplant, was validated in different clinical scenarios, and outperformed a risk score based solely on eGFR, proteinuria and HLA-DSA, not including histological lesions (Table 5). The risk prediction score was also slightly superior to the conventional graft monitoring model based on eGFR and proteinuria assessments in terms of prediction capability; this was further demonstrated by a continuous NRI of 0.228 for the multivariate model compared with the functional model (95% confidence interval 0.174–0.290; *p* < 0.0001). In less-informed datasets, the new algorithm still performed with high accuracy (Table 5) (9).

**TABLE 5:** Risk prediction score performance for iBox when assessed in different clinical scenarios and subpopulations (9).

Risk score performance assessment	Risk model performance (C-statistic)	95% bootstrap percentile CI
Functional and immunological parameters (without histology)	0.79	(0.77–0.81)
Histology diagnoses instead of Banff lesions grading	0.76	(0.74–0.81)
Stable patients (protocol biopsy)	0.81	(0.77–0.86)
Unstable patients (indication biopsy)	0.80	(0.78–0.82)
First year post-transplant	0.77	(0.72–0.81)
After 1 year post-transplant	0.84	(0.82–0.87)
Living donors	0.82	(0.75–0.88)
Deceased donors	0.80	(0.78–0.82)
Highly sensitized recipients	0.80	(0.76–0.84)
Non–highly sensitized recipients	0.81	(0.79–0.83)
Adding transplant baseline characteristics‡	0.81	(0.79–0.83)
Patients with anti-IL-2 receptor induction	0.79	(0.76–0.82)
Patients with antithymocyte globulin induction	0.83	(0.80–0.85)
African American population	0.80	(0.74–0.85)
Non-African American population	0.84	(0.80–0.89)
Recipient blood pressure profile post-transplant	0.80	(0.78–0.82)
Calcineurin inhibitor blood level at time of evaluation	0.81	(0.78–0.83)

CI, confidence interval; eGFR, estimated glomerular filtration rate; IL, interleukin.

The accuracy of the iBox risk score to predict long-term graft failure (9) was confirmed in *post hoc* analyses of data from three RCTs (Table 6) (62–64). Interventions performed in these studies affected the risk score, indicating that iBox adjusts to treatment effects. As the three RCTs did not significantly improve long-term graft outcome in the intervention group, the surrogacy of improvement of the score for predicting improvement of long-term graft survival could not be established directly. However, in the calcineurin inhibitor-free study arm of the CERTITEM study (randomized trial of switch to everolimus vs. cyclosporine continuation) there was a significantly increased risk of developing *dn*DSA in the everolimus group, higher rates of clinical or subclinical rejection, and worse eGFR, all of which were associated with a numerically higher risk of graft failure (5.2 vs. 1.0%). This difference in graft failure failed to reach statistical significance because of low event rates and thus lack of power (64). Post-hoc analysis of the TRANSFORM study (randomized trial of everolimus with reduced exposure calcineurin inhibitor vs. standard-exposure calcineurin inhibitor with mycophenolic acid) (81) indicated that an adapted iBox model (not all parameters were available) confirmed the noninferiority of everolimus with reduced cyclosporine vs. mycophenolic acid with standard cyclosporine for immunosuppression (82). The model projected kidney allograft survival up to 11 years postrandomization. The potential suitability of the iBox risk score as being a surrogate endpoint is further indicated by its general validity, good calibration in RCTs, adjustability over time (and in response to treatment), and its integration of risk factors that are well confirmed in the pathophysiology of (or trajectory toward) graft failure. The evolution after kidney transplantation should be considered as a multidimensional pathophysiology, which could not be identified by looking at one parameter at a time. Importantly, extensive validation through modeling different post-transplant treatment interventions appears to confirm the association between each component of the score and long-term graft failure. For example, the iBox takes account of how a drug might affect kidney function by interfering with renal haemodynamics and eGFR but reducing DSA occurrence. In the context of a clinical trial or immediate therapeutic intervention, each parameter in iBox is individually ranked in terms of the performance, discrimination, and calibration of the risk score.

**TABLE 6:** Clinical trials depicting population characteristics, clinical scenarios and interventions, and prognostic performance of the iBox risk score (62–64).

Study	Trial ID	Design	Clinical scenario	Target population	n	Time post-transplant (y) of risk score evaluation median, IQR	Follow-up time post-transplant (y) median, IQR	Risk score C-stat
CERTITEM (64)	NCT 01079143	Prospective, randomized, open‐label, multicentre trial	Immuno-suppressive drug minimization	Recipients of renal transplants from a living or deceased donor	194	0.94	6.62	0.88
						0.92–0.98	2.82–7.34	
RITUX ERAH (63)	EudraCT 2007-003213-13	Prospective, randomized, multicentre, double-blind, placebo-controlled trial	AMR treatment (pre-existing DSA)	Recipients of renal transplants from a living or deceased donor with diagnosis of aAMR	38	0.74	6.63	0.77
						0.53–1.10	4.03–7.69	
BORTEJECT (62)	NCT 01873157	Prospective, randomized, placebo-controlled, double-blind, single-center trial	AMR treatment (*dn*DSA)	Recipients of renal transplants from a living or deceased donor with post-transplant *dn*DSA detection	44	6.61	7.75	0.94
						4.04–15.41	5.32–16.41	

A, acute/active; AMR, antibody-mediated rejection; dn, *de novo*; DSA, donor-specific antibodies; IQR, interquartile range.

Statistical methodology used in iBox was directly derived from hazard ratio in the Cox analysis; other analyses (e.g., forms of machine learning) were tested but none of the models outperformed Cox, which is widely used in clinical research. The US Food and Drug Administration (FDA) has acknowledged the iBox as a “reasonably likely surrogate endpoint” biomarker to predict 5-years risk of graft failure in kidney transplantation (83). The developers are conducting further modeling to provide additional dimensions, including options for surrogacy, evaluation of its use as an early endpoint in clinical trials, and evaluation of its prognostic ability in subgroup analyses. The developers also plan to make the iBox an open-source platform and are preparing for the 507 drug-development tool qualification process, GDPR compliance, and other aspects of cybersecurity.

Several limitations of the iBox risk score should be noted. Firstly, the method is only useful for prediction of death-censored graft failure: adding death with a functioning graft as a safety endpoint remains necessary. The decision to use the iBox score for predicting death-censored graft failure rather than overall graft failure (including death with a functioning graft) was made because recipient death and loss of graft function have very different causes (3, 4, 71, 84). All-cause graft failure is usually multifactorial and needs a specific design with transplant characteristics, donor characteristics, and factors related to recipient’s comorbidities at time of transplant and thereafter. In sensitivity analyses of the iBox study using competing risk regression models, allograft survival analyses performed in the final iBox model were not affected by competition with patient death.

Next, although the accuracy of the iBox model was maintained irrespective of whether histology was included as individual Banff lesion grades or histology diagnoses, scoring of individual histological lesions included in the composite score is hampered by reproducibility issues and interobserver variability. This limitation is relevant for any scoring system that includes histological parameters, is not specific for the iBox risk score, and needs to be addressed and mitigated in individual clinical trial designs and logistics. In addition, although the iBox score remained accurate across different centers using different methods of tissue typing and HLA antibody profiling, including the MFI of DSA means that this method is impacted by concerns relating to the absolute value of DSA-MFI, which is a semiquantitative rather than quantitative test. This must also be carefully addressed in clinical trial design.

With current evidence, we believe that the approach of multivariate models could be proposed as a surrogate marker for (death-censored) graft failure, since it considers the heterogeneity of causes of graft failure (excluding patient death with a functioning graft). Although it has not yet been shown in randomized trials that improvements in surrogate score actually predict improvements in long-term graft survival, the iBox model is the best-performing and best-validated algorithm to date (Table 6).

## Conclusions

• It is difficult to promote single markers as surrogate endpoints for late graft failure:
  ○ GFR has limitations, since the early course of graft function fails to capture ongoing subclinical disease processes. More sensitive tools are required that reflect heterogeneity in causes of late graft failure.
  ○ Early proteinuria is associated with late graft failure but has not been proposed or tested as a surrogate endpoint in kidney transplantation.
  ○ Combining GFR and proteinuria has a better association with graft failure than either factor separately, but its potential validity as a surrogate endpoint has not been tested.
  ○ Development of *dn*HLA-DSA is associated with graft failure but has not been formally tested or validated as a surrogate endpoint in studies that aim to reduce graft failure caused by AMR. As graft failure also occurs in the absence of AMR, *dn*DSA occurrence is insufficient as a surrogate for late graft failure by causes other than AMR.
• AMR and TCMR are primary endpoints for kidney transplantation clinical trials, which diminishes the need to pursue their validation as surrogate endpoints for late graft failure.
• Death of the recipient with a functioning graft is typically a primary safety endpoint:
  ○ Death of the recipient with a functioning graft is a competing risk for graft failure, but the impact of this competing risk on the accuracy of predictive models is poorly described.
  ○ We recommend not to include recipient death in a surrogate endpoint for late graft failure because of the wide variety of underlying causes of a death observed, different to the causes of graft failure.
• Several composite scores have been proposed and could be useful surrogate endpoints for interventional studies evaluating late graft failure.
  ○ The iBox model is already a well-validated composite score that illustrates the robustness of this integrative approach, although further evaluations are in progress.

### Scientific Advice From the Commitee for Medicinal Products (CHMP) of the European Medicines Agency (EMA) for Human Use Regarding These Conclusions

• The CHMP acknowledged that the ESOT proposes to combine several factors into a single well-validated model as a surrogate endpoint to predict long-term outcome. A surrogate would be expected to fulfill the following three criteria:
  ○ Show biological relationship to the clinical outcome.
  ○ Demonstrate, in epidemiological studies, prognostic value of the surrogate for clinical outcome.
  ○ Provide evidence from clinical trial settings that treatment effect on the surrogate corresponds to effect on clinical outcome.
• The CHMP noted that ESOT introduces the iBox model (9) to predict long-term kidney graft failure at 3, 5, and 7 years, based on the following factors:
  ○ Time from transplant to risk evaluation after transplantation.
  ○ eGFR.
  ○ Proteinuria.
  ○ Banff IFTA grade, g + ptc, cg, and i + t scores.
  ○ MFI of donor-specific HLA antibodies.
• Based on ESOT´s position and the publication by Loupy et al. (9), the score appears to be designed as a risk calculation score and validated as such in separate cohorts. As such, the iBox score could provide an important contribution to the stratification of participants of clinical trials of transplantation.
• It is not clear if the third criterion above has been fulfilled, i.e., that treatment effect measured via iBox translates into corresponding effect on clinical outcome, i.e., graft failure. Furthermore, the following issues need to be addressed:
  ○ The statistical model and iBox algorithm were not presented and the relative contribution of each factor of the model was not evident; several factors of the iBox are also interrelated, e.g., histological diagnosis and the various histological lesions.
  ○ “Time from transplant” is an important prognostic marker but is never affected by therapy, therefore it cannot predict the effect of therapy on clinical outcome.
  ○ Outcome of iBox included death-censored graft failure, which is not a robust and favored clinical endpoint to show surrogacy, as there are several limitations in using the score without additional sensitivity analyses.
• ESOT showed the correlation of each variable in the final iBox model to death-censored functional outcome, a density plot of iBox evaluations post transplantation and the hazard ratio of each factor of the model.
  ○ Sensitivity analysis of the iBox indicate that graft survival analysis was not affected by competition with patient death.
  ○ ESOT noted that all-cause graft failure was multifactorial, with very different risk factors than death-censored graft failure, where grafts from patients who died with a functioning graft, were defined as functional grafts in the model.
  ○ However, ESOT acknowledged the concern regarding the importance of all-cause mortality in clinical trials of kidney transplantation for regulatory purposes and proposed to include this as part of safety or composite endpoints.
  ○ ESOT outlined the plans to further explore these issues with the FDA, including the preparation for a Drug Development Tool (DDT) qualification process.
• For the time being iBox is not qualified as a surrogate endpoint for regulatory purposes and thus cannot be proposed *a priori* to be used in clinical practice to guide decision making.
  ○ Based on the high-level data provided, CHMP notes that there are still certain limitations in applying the iBox score for regulatory purposes: the applicability of this score seems limited to certain determinants of kidney graft and the death-censored functional aspect.
  ○ A formal EMA Qualification of Novel Methodologies procedure for the finalized iBox as a surrogate marker would be very relevant way forward and is recommended.
